# Hyperbaric oxygen therapy modulates serum OPG/RANKL in femoral head necrosis patients

**DOI:** 10.1080/14756366.2017.1302440

**Published:** 2017-04-07

**Authors:** Giuliano Vezzani, Silvia Quartesan, Pasqua Cancellara, Enrico Camporesi, Devanand Mangar, Thomas Bernasek, Prachiti Dalvi, Zhongjin Yang, Antonio Paoli, Alex Rizzato, Gerardo Bosco

**Affiliations:** a Department of Biomedical Sciences, Physiological Laboratory, University of Padova, Padova, Italy;; b Anesthesia, Tampa General Hospital; TEAM Health Anesthesia Research Institute, Tampa, FL, USA;; c Florida Orthopedic Institute, Tampa, FL, USA;; d The Institute for Human Performance, SUNY Upstate Medical University, Syracuse, NY, USA

**Keywords:** Avascular necrosis of femoral head, bone remodeling, hyperbaric oxygen therapy, serum osteoprotegerin, serum receptor activator of NF-kB Ligand

## Abstract

Hyperbaric oxygen therapy (HBOT) has beneficial effects on avascular necrosis of femoral head (ANFH), but its mechanism of action is still unclear. We investigated if HBOT upregulates serum osteoprotegerin (OPG) and/or inhibits osteoclast activation. 23 patients with unilateral ANFH at stage I, II and III consented to the study: the patients received standard HBOT. Serum OPG levels were obtained at the beginning of HBOT (T0), after 15 sessions (T1), 30 sessions (T2), after a 30-day break (T3), and after 60 sessions (T4). Magnetic resonance imaging (MRI) was obtained at T0 and about one year from the end of HBO treatments. Lesion size was compared between pre- and post-HBOT. 19 patients completed the study. HBOT reduced pain symptoms in all patients. HBOT significantly reduced lesion size in all stage I and II patients and in 2 of 11 stage III patients. HBOT increased serum OPG levels but receptor activator of nuclear factor kappa-B ligand (RANKL) levels did not change.

## Introduction

Avascular necrosis of femoral head (ANFH) is a pathologic process that results from compromised blood supply to the bone structure. Etiological factors of ANFH include trauma, infection, and excessive steroid use^1^. Femoral head ischemia results in the collapse of the necrotic segment, as a result of an imbalanced bone remodeling process. Bone remodeling (or bone metabolism) is a lifelong process that basically involves two types of cells: osteoblasts, responsible for bone formation, and osteoclasts, responsible for bone resorption. Their activity is finely tuned by osteoprotegerin (OPG), the receptor activator of NF-kB Ligand (RANKL), and the receptor activator of NF-kB (RANK) system. Any perturbation affecting this system may shift the bone remodeling balance toward bone resorption, which eventually leads to pathological states, such as the degradation and collapse of the femoral head^2^.

RANK is a transmembrane protein of osteoclasts and their hematopoietic precursor (circulating monocytes). RANKL, the only ligand of RANK, binds to the extracellular portion of RANK inducing osteoclast differentiation, activation, prolonging their life and strengthening their adhesion to bone surfaces. In detail, activated RANK transduces intracellular signals by recruiting TNF receptor-associated factor 6 (TRAF6), which activates transcription factors, such as nuclear factor kappa B (NF-kB).

RANKL is produced by many cell types, including osteoblasts, endothelial cells, and active T lymphocytes; it can be present in cell-bound and soluble forms (soluble RANKL, s-RANKL). OPG, the soluble decoy receptor for receptor activation of nuclear factor-kappaB ligand (RANKL), inhibits RANKL binding to RANK and prevents osteoclastogenesis and bone resorption. OPG is produced by osteoblasts, as well as by other cell types, including peripheral blood lymphocytes^3^^,^^4^.

The equilibrium of this system is regulated by many osteotrophic hormones and cytokines, which either reduce or increase the OPG/RANKL ratio. Any change that affects the molecular triad – OPG/RANKL/RANK – results in an alteration of bone remodeling that, if not duly controlled, may lead to the development of skeletal abnormalities characterized by decreased (osteoporosis) or increased (osteopetrosis) bone mass^5–10^. Interestingly, the ratio of RANKL to OPG in sera and necrotic femoral head reverses changes in steroid-induced osteonecrosis of the femoral head in rats^11^. Furthermore, Grimaud et al.^12^ evidenced that in severe osteolysis, serum OPG protein increased but serum RANKL protein decreased. These studies, together, suggest that serum OPG/RANKL is involved in bone remodeling and in bone diseases. Non-operative treatment might delay femoral head collapse, avoiding joint replacement procedure^12^^,^^13^. Regional ischemia causes joint structure hypoxia, which is a common feature of ANFH. By providing 100% oxygen at elevated atmospheric pressure, hyperbaric oxygenation (HBO) increases the partial pressure of oxygen in plasma and in tissues. In turn, this allows the extra oxygen to be diffused or transported to the body tissues. As an adjunctive therapy, HBOT has been reported to improve the outcomes in patients suffering from bone diseases^13^ and femoral head necrosis^14–16^. HBOT significantly alleviated the pain and improved range of motion in ANFH patients. In addition, recent works reveal that hyperbaric oxygenation may also accelerate osteoblast differentiation and suppress osteoclasts genesis-activation, shifting the balance between bone formation and bone resorption in a direction that promotes regeneration^17^^,^^18^. However, less is known about the molecular mechanism underlying its effectiveness. The present study was designed to examine our hypotheses that (1) HBOT improves the affected joints’ bony structure in ANFH; and (2) the beneficial effect of HBO may be via modulating OPG/RANK/RANKL system.

## Materials and methods

### Patient selection

This study was approved by the Institutional Ethics Committee of the University of Padova (Biomedical Science Department) and was conducted in accordance with the ethical standards of the Helsinki Declaration. This prospective study involved 23 patients with unilateral femoral head necrosis, including post-traumatic femoral head necrosis (PT-FHN), post steroid therapy femoral head necrosis (PS-FHN), femoral condyles necrosis (FCN) and other aseptic bone necrosis (OABN). Patients with other diseases were excluded from the patient population. Informed consent was obtained from all patients before the start of the study. Every patient had a plain X-ray of the hip in 2 projections (anterior and lateral) and then had magnetic resonance imaging (MRI) to stage their pathology according to the Ficat classification^19^. Patients were exposed to breathing 100% oxygen at 2.4 atmospheres absolute in a multi-place pressure chamber for 90 min using an overboard demand regulator while breathing through an oral-nasal mask for 5 days a week as described in our previous study^15^. Subsequent MRIs were performed at various times: T0 (beginning of HBOT), T1 (after 15 HBOT sessions), T2 (after 30 HBOT sessions), T3 (beginning of the second HBOT cycle after a 30-day break), T4 (end of the second HBOT cycle). The endpoint of the study was a normal MRI one year after the end of second cycle. According to MRI criteria established by Vande Berg^20^, the subchondral lesion was 4 mm or more thick and/or 12.5 mm or more long. The endpoint was a comparison between immediate post-treatment and pretreatment MRI, sufficient to assess the efficacy of HBOT. Visual Analog Scale (VAS) pain scores were obtained through a standardized questionnaire before the beginning and the end of HBOT treatment. Pain score was used to define the improvement, or aggravation of the pain after HBOT treatment. The HBOT protocol and OPG and RANKL assay are detailed in Appendix A.

### Statistical analysis

Data were analyzed by a qualified statistician using Prism software for Mac, version 6.0c (GraphPad Software, San Diego, CA). Obtained results were presented as means ± SEM. One-way repeated measure ANOVA was performed to evaluate the level of serum OPG and RANKL between pretreatment and post-treatment of HBO. We used the Shapiro–Wilk test to ensure normal distribution and homogeneity of variance of our data (*p* ≫ .05). The value of *p* < .05 was considered statistically significant.

## Results

The demographic features of the study population are presented in Table 1. Mean age was 54.2 ± 10.1 years. There are 11 female and 12 male patients, respectively. An equal number of patients had stage I, II and III osteonecrosis of the femoral head. 19 of 23 subjects completed the experimental protocol.

**Table 1.** Details of the demographic features of the patients and the levels of severity of the disease.

		Ficat stage			Sex (*n*)	
Patients (*n*)	Age (years)	I	II	III	Male	Female
23	54.2 ± 10.1	1	7	15	12	11
Etiology						
FHN Ficat stage (*n*)		PT-FHN Ficat stage (*n*)			PS-FHN Ficat stage (*n*)	OABN Ficat stage (*n*)
II	III	I	II	III	III	III
6	9	1	1	1	3	2

PTFHN: post-traumatic femoral head necrosis; PSTFHN: post steroid therapy femoral head necrosis; FCN: femoral condyles necrosis; OABN: other aseptic bone necrosis.

As shown in Table 2, all patients with stage I and II ANFH returned to normal anatomy on MRI as compared with pretreatment. Improvement of patients with stage III ANFH was detectable only in two patients from imaging analysis and only 3 of 11 subjects had relief of symptoms. HBOT significantly reduced lesion size and pain score in all stage I and II patients. However, reduced lesion sizes and pain scores were only seen in 18% and 27% of stage III patients, respectively.

**Table 2.** MRI-evidenced improvement after HBOT in patients with ANFH.

	Total patients (*n*)	Improvement after HBO treatment	Aggravation after HBO treatment	Unchanged	Improvement in VAS pain score	Aggravation in VAS pain score
Ficat I	1	1	0	0	1	0
Ficat II	7	7	0	0	7	0
Ficat III	11	2	3	6	3	4

HBO: hyperbaric oxygenation.

**Figure 1.**
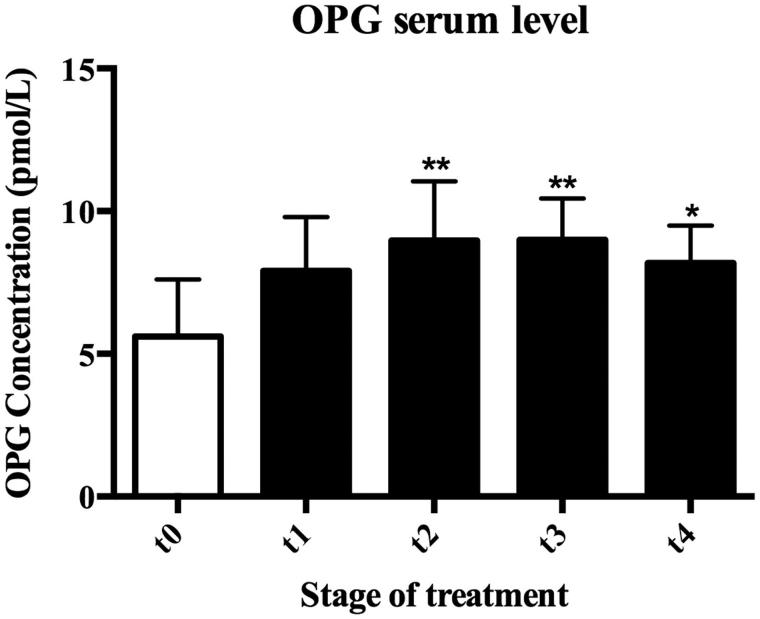
Effects of HBOT on serum OPG levels in ANFH patients. HBO therapy significantly increased OPG serum concentrations throughout the whole experiment. OPG concentrations were 5.61 ± 1.99 pmol/L, 7.90 ± 1.90 pmol/L, 8.97 ± 2.07 pmol/L (*p* < .01), 8.99 ± 1.46 pmol/L (*p* < .01), and 8.18 ± 1.31 pmol/L (*p* < .05) at T0, T1, T2, T3, and T4, respectively. T0: Baseline, T1: after 12 HBO, T2: after 30 HBO, T3: after 45 HBO, and T4: after 60 HBO. *p < .05; **p < .01

In our study, ELISA assays were performed on serum samples of patients in order to determine OPG and soluble RANKL concentrations. Obtained results show, in almost all subjects, increased levels of plasmatic OPG above control level in response to HBO therapy. In detail, OPG levels, were 5.61 ± 1.99 pmol/L in T0, 7.90 ± 1.9 pmol/L in T1, 8.97 ± 2.07 pmol/L (*p* < .05) in T2, 8.99 ± 1.46 pmol/L (*p* < .01) in T3, and 8.18 ± 1.31 pmol/L (*p* < .05) in T4 (Figure 1).

**Figure 2.**
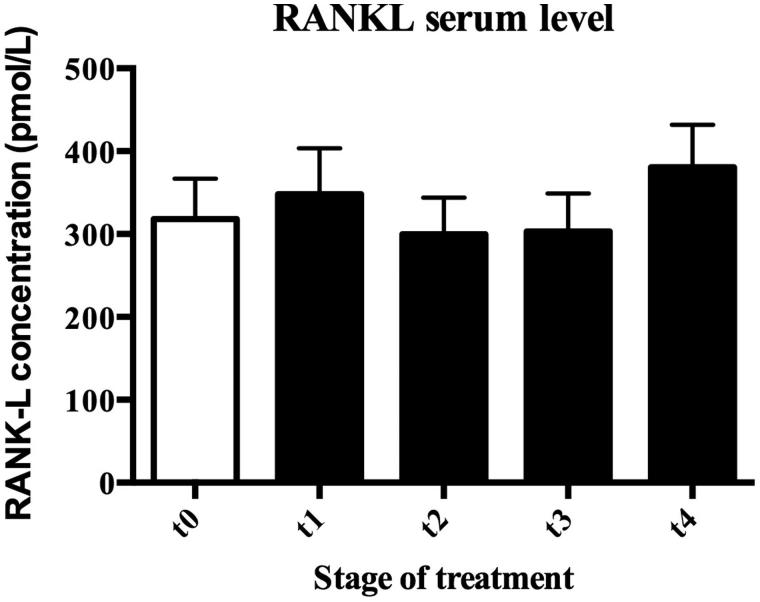
Effect of HBOT on serum RANKL level in ANFH patients. There was no significant change in serum RANK-L levels during HBO treatment, being 318.2 ± 48.66 pmol/L, 347.7 ± 155.40 pmol/L, 299.7 ± 44.36 pmol/L, 303.2 ± 45.89 pmol/L, and 380.5 ± 51.41 pmol/L at T0, T1, T2, T3, and T4, respectively. T0: Baseline, T1: after 12 HBO, T2: after 30 HBO, T3: after 45 HBO, and T4: after 60 HBO.

Contrastingly, no significant change in serum RANKL levels was appreciated during HBO treatment, being 318.20 ± 48, 66 pmol/L in T0, 347.70 ± 155.40 pmol/L in T1, 299.70 ± 44.36 pmol/L in T2, 303.20 ± 45.89 pmol/L in T3, and 380.50 ± 51.41 pmol/in T4, (Figure 2).

Overall, our findings suggest that HBOT promotes bone regeneration influencing the plasmatic OPG/RANK/RANKL system, specifically by increasing OPG serum level significantly.

As shown in Tables 1 and 2, HBOT significantly improves joint structure as evidenced by MRI in all Ficat I and II patient and in some Ficat III patients. HBOT also eliminates the pain in all Ficat I and II patients and reduced the pain in some of Ficat III patients.

## Discussion

The main findings of this study are that (1) HBOT reduced lesion size and improved clinic symptoms in ANFH patients; (2) HBOT reduced joint pain and improved joint structure as evidenced by MRIs; and (3) HBOT affects OPG expression. We have previously reported the beneficial effect of HBOT in patients with stage II ANFH^15^. The present study provides further evidence to validate the usefulness of HBOT in the management of ANFH.

The natural duration of femoral head collapse is ranged from 2 months to 3 years (mean 1.4 years) in stage III ANFH and from 4 months to 12 years (mean 6.2 years) in stage II^21^. Koren et al. used HBO to treat patients with stage I and II ANFH and found that the stage I patients responded to HBOT much better than stage II patients^22^. Our results also showed an inverse relationship between the effect of HBOT and the stages of ANFH. When the femoral head collapses, HBOT might not be able to significantly improve the outcome. Koren et al. suggested that after HBO therapy, the survival of intact joint was 93% in stage I and 90% in stage II after 11.1 ± 5.1 years follow up^22^. In our study, the rate of joint replacement in stage II patients was lower than that in stage III patients. Therefore, in order to achieve better therapeutic effect, HBOT should be applied in patients with early stage (pre-collapse) ANFH.

Osteoprotegerin (OPG), Receptor Activator of Nuclear Factor Kappa-B (RANK), and RANK ligand (RANKL) regulate the balance between osteoclasts–osteoblasts. The expression of these genes affects the maturation and function of osteoblasts–osteoclasts and bone remodeling. Samara et al. investigated the molecular pathways leading to ANFH by studying the expression profile of OPG, RANK, and RANKL genes. Interestingly, they found that while mRNA and protein levels of each gene are comparable in normal tissue samples, most necrotic samples had higher or very low OPG and RANKL protein levels compared to their respective mRNA. In particular, the difference between normal and necrotic tissues is seen at the mRNA level, which suggests the existence of a post-translational control especially in necrotic tissue^23^.

Wang et al. investigated the expression levels of OPG and RANKL mRNAs in bone tissues of the femoral head of the patients suffering glucocorticoid-induced osteonecrosis (ONFH) of the femoral head^24^. They found lower expression level of OPG mRNA and higher expression level of RANKL mRNA in affected area of ONFH patients than in a corresponding area of control group. The OPG mRNA/RANKL mRNA ratio in the experiment group was significantly lower than that in the control group. These results demonstrate the relationship between glucocorticoid-induced femoral head necrosis and the expression levels of OPG mRNA/RANKL mRNA in bone tissues. Our study provides further evidence that changed serum OPG and RANKL levels, in terms of proteins, are involved in the development of ANFH.

HBO has been documented by the Undersea and Hyperbaric Medicine Society to be beneficial for 15 different diseases^25^. HBO therapy involves the administration of 100% oxygen at pressures higher than atmospheric pressure to the entire body. As a higher oxygen concentration is dissolved in the blood and delivered to bone tissue, the oxygen is made available to ischemic bone cells^14^^,^^26^. Meanwhile, because of its vasoconstrictive effect, HBOT reduces marrow edema, improving venous drainage and microcirculation^27^. Indeed, angiogenesis induced by HBOT contributes to the recovery of an osteonecrotic femoral head^28^. On the other hand, we cannot overlook the evidence that this treatment increases bone formation and suppresses bone resorption, an effect that may not be only due to an improved local circulation^11^. Thus, HBO therapy can improve the natural progression of ANFH, but not all mechanisms involved are completely understood.

Our study has also suggested, for the first time, that HBO’s therapeutic effect is associated with increased serum OPG levels. Yano et al. reported that the serum concentration of OPG increased with age in both healthy men and women, and was significantly higher in postmenopausal women with osteoporosis than in age-matched controls. Within the osteoporotic group, serum OPG concentrations were higher in patients with low bone mass. The authors suggested that circulating OPG levels are regulated by age-related factor(s) and that the increased serum concentration may reflect a compensatory response to enhanced osteoclastic bone resorption and resultant bone loss rather than form osteoporosis^28^. In the present study, the changes in serum OPG reflected the response of the patients to HBO treatment. Notably, this response was not counteracted by different soluble RANKL concentrations. Upregulation of RANKL, RANK mRNA, and down-regulation of OPG mRNA in tissue samples have been detected in patients with ANFH^9^^,^^29–33^.

In the present study, we show that HBOT significantly increases serum OPG and this level is maintained throughout the experiment. This seems to suggest that HBOT, by upregulating OPG production, may reduce osteoclast activation and formation. In particular, maximum OPG levels have been measured in T2 (8.97 ± 2.07 pmol/L), at the end of the first cycle of therapy. It is significantly higher (*p* = .009) when compared to pre-HBOT OPG levels (T0: 5.610 ± 1.99 pmol/L) and this trend seems to be maintained 30 days after the therapy was stopped (T3: 8.99 ± 1.46 pmol/L). At the end of the second cycle of HBOT, OPG concentration in serum is still higher than T0 (*p* = 0.04), but a little lower than at the beginning of the second cycle of HBOT (T3). On the contrary, RANKL values seem to decrease in response to HBO treatments until T3 (T0: 318.20 ± 48.66, T3: 303.20 ± 45.89 pmol/L) in most patients. At the end of the second cycle (T4: 380.50 ± 51.41 pmol/L) RANKL concentrations remain almost constant and return to the initial values only in a few subjects (Figure 2). The variability measured is not significant.

We can speculate that hyperbaric oxygenation is most effective when performed in the earlier stages and has the potential to influence serum OPG concentration significantly. We can also hypothesize that the second series of HBO may act to reinforce this response and to sustain the positive clinical outcomes over the years; alternatively, it may only be redundant.

Though our study is limited by small sample size, overall, our findings suggest that HBO therapy influences OPG and RANKL plasmatic values shifting their balance in a direction that promotes bone regeneration.

Moreover, our work confirms that serum OPG and RANKL measurements are noninvasive tools useful for assessing bone remodeling in patients with metabolic bone disease. However, as previously explained, the serum OPG/RANKL ratio is influenced by multiple factors, including immune function^34–37^. Therefore, understanding the clinical utility of serum OPG and RANKL measurements as markers of disease activity requires further investigation.

In conclusion, HBOT is a safe and effective therapeutic modality in managing patients with ANFH. The beneficial effect of HBOT may be via upregulating serum OPG production to influence the bone remodeling process in ANFH.

## Appendix A

### HBOT protocol

HBOT protocol has been described in our previous study^15^. Patients were exposed to HBO inside a multi-place hyperbaric chamber (Galeazzi, Bergamo, Italy) with compressed oxygen at 2.4 ATA for 90 min, comprising a period of 60 min when the patient was continuously exposed to 2.4 ATA without interruption. Each patient was provided with a well-sealed breathing mask, from which he or she received 100% oxygen. Oxygen concentration in the mask was measured every 5 minutes to ensure adequacy of the gas supply and the ability to provide a tight seal around the face. Patients received HBO once a day, from Monday to Friday, for 6 weeks (30 HBOT sessions in total). After a one-month break, they received 30 more HBOT. Overall, these patients received 60 HBOT sessions over 12 months.

### OPG and RANKL assay

Meanwhile, blood samples were obtained at T0, T1, T2, T3, and T4. Serum concentration of OPG and RANKL were analyzed with ELISA kits according to manufacturing instructions (Total RANKL Human ELISA kit and Osteoprotegerin Human ELISA kit produced by Bio Vendor LLC, Asheville, NC 28806). Limit of detection was 0.03 pmol/L.
